# Supplementary figures and images for: Understanding implementation, adoption, and acceptability of the WHO package of essential noncommunicable (PEN) disease interventions in FIJI: Evidence for scale-up
Source: PLOS Glob Public Health. 2025 Apr 21;5(4):e0004344. doi: 10.1371/journal.pgph.0004344 (PMC12011304; doi:10.1371/journal.pgph.0004344)

S1 Appendix Logic model used for planning phase of evaluation


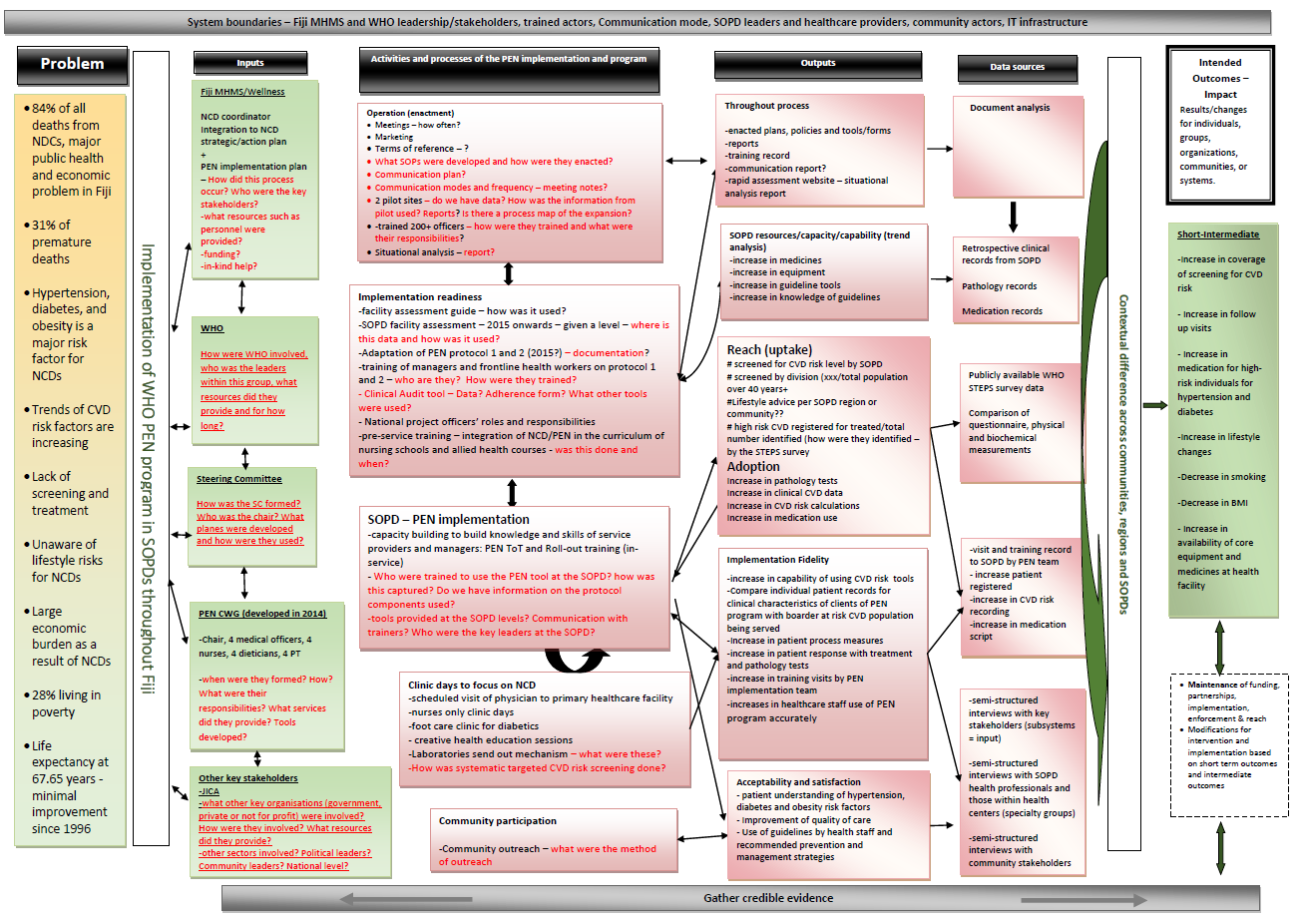

Supplement: S1 Appendix — (DOCX) [file pgph.0004344.s001.docx]

S3 Appendix Mind Map


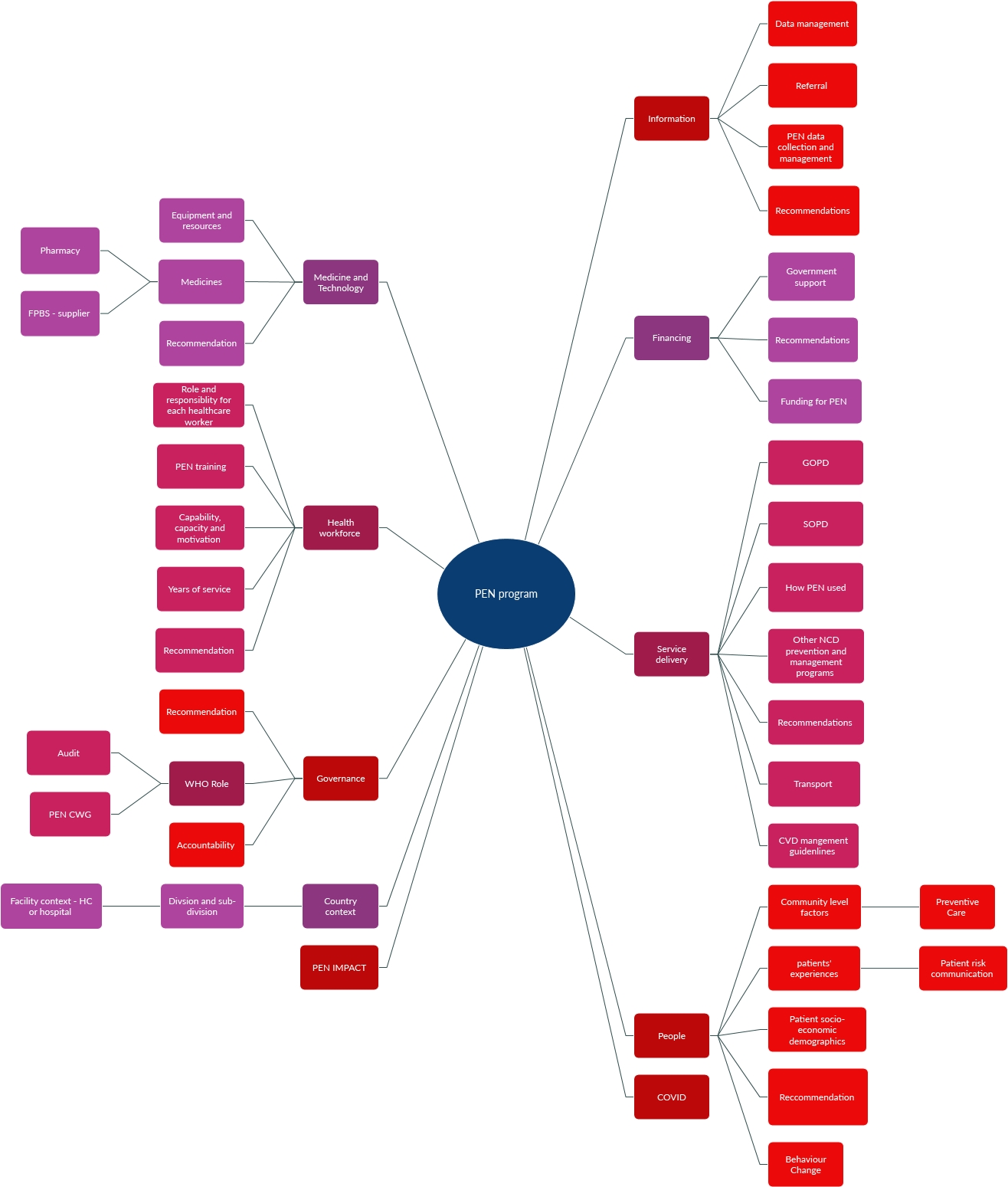

Supplement: S3 Appendix — (DOCX) [file pgph.0004344.s003.docx]
