# Supplementary material for: Understanding implementation, adoption, and acceptability of the WHO package of essential noncommunicable (PEN) disease interventions in FIJI: Evidence for scale-up
Source: PLOS Glob Public Health. 2025 Apr 21;5(4):e0004344. doi: 10.1371/journal.pgph.0004344 (PMC12011304; doi:10.1371/journal.pgph.0004344)
Supplement: S2 Appendix — (DOCX) [file pgph.0004344.s002.docx]

S2 Appendix Healthcare Worker and Patient Interview Guides

Ask respondent(s) to introduce themselves and talk a little about themselves and their current role.

| Area of enquiry | Broad interview question | Possible probing questions |
| --- | --- | --- |
| **Knowledge of Innovation** – Package of Essential Non (PEN) Communicable Disease Interventions | Can you tell us how you 1^st^ heard about the PEN program?  How was the PEN program introduced to your facility?  How did the program compare to what you already were using for prevention and management of CVD and diabetes? | What were you told was the objective of the program?  What did you think when it was introduced to your facility? |
| **Knowledge of implementation methods** | What were you told would be involved to use the PEN program? |  |
| **Knowledge – training provided by implementers** | How was the training of the program provided? | Face to face  Email, etc  Who were the training provided by? |
|  | Who at the facility was provided the training to use the PEN guidelines and tools? | Did you discuss with others that were trained what they thought of the program and use of the tools? Did they understand how to use the guidelines for prevention of CVD and diabetes and management? |
|  | How did the implementers check up on how you were doing? And if additional help was needed? | Initial training  Subsequent training  How often did the implementers come to your facility to assist with the use of the program? |
|  | Did you feel after the training was provided that you had the right skill set and capability to use the PEN tools? Why? |  |
| **Facility (organisational level)** | How was your facility assessed to see if you had the proper equipment and medicines to use the PEN Guidelines and Tools? | -was there a site assessment performed?  -follow-up after site assessment conducted.  -where was the data stored and how was it used? |
|  | How was equipment provided to you? Did you and others at the facility need training on using the equipment? | If you needed training, how was it provided? |
|  | How were medicines supplied to you? |  |
|  | How did the PEN program fit into your everyday routine care? |  |
|  | Was there a re-organisation of the staff at your facility to allow for time to use the PEN tools? If yes, what was the process? If no, how do you think this affected the use of the tool? |  |
|  | What were some of the adaptions of the PEN tools that took place based on your facility governance, resources, and staff? |  |
| **Use of the PEN program (adoption)** | Can you tell us how often you used the PEN tools to assess risk and treat patients? | - Per week? Over time? - **%** |
|  | How did you use the PEN tools? | - Can you remember the last patient you used it on? |
|  | How has it integrated into your routine care? |  |
|  | How did patients react to hearing about their risk for CVD? Diabetes? |  |
|  | Are more patients aware of their risk? How are patients working to prevent or treat their risk for CVD and/or Diabetes? |  |
| **Barriers and enablers to the use to the PEN program** | What do you think is needed to have the PEN program used on a regular basis at the SOPD facility? |  |
|  | How do you think it can be used with patients to have most impact? | - Reduce risk of CVD and diabetes - Decrease BMI - Active lifestyle - Change diet - medicines |
|  | What has worked with the PEN program? |  |
| **Recommendation for scale-up** | Please tell us your recommendations for how we can implement more effectively at your facility and the primary health care level. |  |

**Patient Interview Guide**

Opening question

*To begin with, I’d like you to think about the healthcare services that is provided to you and what you think of the experience especially in relation to your heart health and diabetes.*

Follow up questions

*We are now going to ask a series of questions about how your heart health and blood sugar is cared for:*

| Area of enquiry | Question |
| --- | --- |
| Healthcare experience | **These questions are about your perspective on your healthcare experience.**  Do you feel that your health needs are adequately met in the time you spent with the doctor?   - What are some of the good/bad things about your health care? - Why did you go visit your doctor? What were your health needs? - Do you discuss heart disease, such as high blood pressure and cholesterol with the nurse or doctor or any health care worker? If yes, is this discussed regularly at your visit? |
| Management of Heart disease | How does the doctor or other health care worker provide care for the health of your heart?   - Blood pressure, cholesterol, blood sugar, smoking, diet, or exercise   Have you had any discussions with your doctor about heart disease risk? If yes, what type of discussions did you have?   - Treatment/mediations, diet, smoking, exercise? |
| PEN program | Have you heard about the program named the PEN program at your clinic? It stands for Package of Essential Interventions for Non-communicable Interventions? If yes, what have you heard?   - Has your doctor or healthcare worker used any recommendations from the tool?   [if they have not heard about the PEN program, show them pictures of what it is and how healthcare workers should be using it for the patients]   - It helps the doctor or healthcare worker to help you to prevent or manage your heard health risk and diabetes risk by the following: - Lifestyle changes - use of medicines - involvement of other care providers |
| Shared decision making and risk communication | How did your doctor explain your risk of heart disease to you?   - If yes, how does he explain? Are there any pictures that he shows you? - Do you member if a % risk was given to you by the doctor? If yes, what did that mean to you? Did you understand it?   Did the doctor tell you that you need to be on medication for heart disease? If yes, how did you feel about starting new treatment? |
|  | How would you describe your chance/risk of having heart disease or high blood sugar that causes diabetes?   - Would you say you are low risk, medium risk, or high risk? Why |
| Actions | Does your doctor or health care worker recommend any changes to reduce your heart risk?  -More exercise  -Quit smoking or drinking  -Eat better  - reduce your weight by lifestyle changes  Are you planning to make any lifestyle changes? |
