# Supplementary material for: Understanding implementation, adoption, and acceptability of the WHO package of essential noncommunicable (PEN) disease interventions in FIJI: Evidence for scale-up
Source: PLOS Glob Public Health. 2025 Apr 21;5(4):e0004344. doi: 10.1371/journal.pgph.0004344 (PMC12011304; doi:10.1371/journal.pgph.0004344)
Supplement: S4 Appendix — (DOCX) [file pgph.0004344.s004.docx]

S4 Appendix

|  | **Case 1** | | **Case 2** | | **Case 3** | | **Case 4** | | **Case 5** | | **Case 6** | | **Case 7** | |
| --- | --- | --- | --- | --- | --- | --- | --- | --- | --- | --- | --- | --- | --- | --- |
| **Division** | **Western** | | **Western** | | **Northern** | | **Central** | | **Central** | | **Western** | | **Eastern** | |
|  | **Enablers** | **challenges** | **Enablers** | **challenges** | **Enablers** | **challenges** | **Enablers** | **challenges** | **Enablers** | **challenges** | **Enablers** | **challenges** | **Enablers** | **challenges** |
| **Health Workforce** | Presence of some health workers utilizing PEN  *Few staff trained.  *Some staff who were not formally trained on PEN has heard about it in medical school or School of Nursing | *Shortage of workforce  *High turnover of medical officers  *No dietician and physiotherapist  *Some staff NOT trained for PEN Model | * Facility has doctors, nurses, dietician and physiotherapist  *Doctor trained on use of PEN model  *Some trained HCWs provide on-the-job training on PEN for new ones | *Some staff NOT trained for PEN Model  *High staff turnover  *Shortage of workforce | *Facility has doctors, nurses, physiotherapist, dietician, pharmacist, radiologist and lab technician  *Has a managerial nurse and an experienced, long-time Physiotherapist   *Motivated and capable doctors   *Some staff trained PEN workshop | *High turnover of Medical Officers  *High workload  *Some staff NOT trained for PEN Model | * Facility has doctor, nurses, dietician, physiotherapist and pharm assistant.  *Few staff trained PEN workshop | *Some staff NOT trained for PEN Model  *Shortage of workforce | *Facility has doctors, nurses, physiotherapist, dietician, pharmacist, and nurse practitioner.  *Dietitian based at Health Centre | *Some staff are not trained on PEN, especially new SOPD staff  *High turnover of staff   *Staff shortage | * Facility has doctor, nurses, dietician, physiotherapist and pharm assistant.  *MO trained during Public Health post-graduate training  *Only dietitian trained on motivational Interview | *Some staff are not trained on PEN, so unable to support the PEN model implementation  *Shortage of health workers  *No designated pharmacist | *Doctor, dietician and nurse trained on PEN | *Transfer of trained staff from HC interrupts the continuity of PEN programme if new staff is untrained.  *Some staff are not trained on PEN and they are not able to support the PEN model implementation |
|  |  |  |  |  |  |  |  |  |  |  |  |  |  |  |
| **Medicine** | *Availability of some essential medication for DM and HTN patients | *Delayed delivery and occasional prolonged out-of-stock of essential DM and HTN medication  *Sufficient quantity of the most used medications (e.g. metformin) are hardly supplied  *Some patients are not able to purchase medication out-of-pocket | *Availability of essential medications for DM and HTN patients almost always  *No limitation with essential medicines except if it is a national issue | Occasional out-of-stock of essential medicines *(only when it is a national issue)* | *Availability of some essential medication for DM and HTN patients | *Delayed delivery and occasional prolonged out-of-stock of essential DM and HTN medications  *Sufficient quantity of the most used medications (e.g. metformin) are hardly supplied  *Sometimes, medications are dispense based on what is available instead of what is prescribed - as this is considered a better option for patient than not using any (due to financial challenge) | *Availability of essential medications for DM and HTN patients almost always  *No limitation with essential medicines except if it is a national issue | Occasional out-of-stock of essential medicines (only when it is a national issue) | *No major issues with medication supply for blood pressure or diabetes  *MHMS provides free or highly discounted medications through a welfare service for patient who can't afford them | *Some essential medications are sometimes out of stock.  *Some patients are not able to purchase medication out-of-pocket | *Availability of essential medications for DM and HTN patients almost always  *No limitation with essential medicines except if it is a national issue | *Out-of-stock of essential medicines (only when it is a national issue) | *Availability of some essential medication for DM and HTN patients | *delayed delivery and occasional prolonged out-of-stock of essential DM and HTN medication that are dependent on supply chain processes from FPBS (e.g. out-of-stock for as long as 6 months)   *Sufficient quantity of the most used medications (e.g. metformin) are hardly supplied |
|  |  |  |  |  |  |  |  |  |  |  |  |  |  |  |
| **Equipment and Technology** | Availability of some basic equipment such as glucometer, blood pressure machine  *Donation of new BP apparatus by individuals and private organizations | Limitation on core equipment especially glucose strips and functional blood pressure machine  *Challenges with repairing or replacing faulty BP apparatus  *Cholesterol strips are not usually available | *Availability of essential technology and equipment needed for PEN  *WHO donated basic equipment for PEN | No limitation with essential technology and equipment | Availability of some basic equipment such as glucometer, blood pressure machine | Limitation on core equipment especially glucose strips and functional blood pressure machine | *Availability of essential technology and equipment needed for PEN  *Sister-in-charge (senior nurse) can get replacement for faulty equipment | *Some basic equipment are shared across departments  *Facility experiences shortage of strips foe blood glucose monitoring | *No major issues with basic equipment required for PEN implementation  *Digital BP apparatus malfunctioning intermittently | *Glucose strips can be an issue   *Due to lack of cholesterol strip, patient are sent to the lab for tests - some of the results don't get back to SOPD (because they are manually dispatched) | *Availability of essential technology and equipment needed for PEN | *Glucose strips can be an issue  *Due to lack of basic equipment and supplies, for cholesterol, patient are sent to the lab for tests - some of the results don't get back to SOPD (because they are manually dispatched) | Availability of some basic equipment such as glucometer, blood pressure machine | Limitation on core equipment especially glucose strips and functional blood pressure machine   *Challenges with repairing or replacing faulty BP apparatus  *Cholesterol strips are not usually available |
| **Service delivery** | * Despite not being formally trained, some staff has learnt how to use PEN Protocol through observation  *Awareness session within the community enabled early detection and prompt treatment  *Patients have access to counselling that support lifestyle modification | *Time constraints and shortage of staff affects quality of care   *Nurse understands risk calculation concept but don't feel comfortable calculating it  *Limited risk communication by doctor to patient due to insufficient time  *No effective SNAP programme due to lack of a dietician  *Prevention services to the community (e.g. CVD risk screening) are limited due to factors such as lack of transportation  *Lack of dietician limits education on heart-healthy diet *Significant issues with performing roles in the community due to lack of transportation) | *PEN protocol implemented within the GOPD | *Lack of separate facility and sufficient staff limits PEN use  *High workload limits the use of PEN guidelines  *Time constraints limit services for counselling on diet and physiotherapy activities  *Limited days for implementing PEN model due to insufficient staff  *Patients do get lost to follow-up when the workload is high for HCWs Retention is about 70% (i.e. 30% LTFU) | *SOPD Nurse and MO use Protocol 1   *Physiotherapist uses PEN Protocol 2  Dietician utilized patients' colour code/CVD risk classification to individualize dietary management  PEN Model has helped dietician to counsel patient better  *PEN Model helps SOPD patient to understand their condition and how they are being managed  *Physiotherapist is able to prescribe exercise to the patients based on CVS risk classification | *MO does not follow Protocol 1 completely (especially with increased workload)  *Dietician's services were interrupted in SOPD as the only dietician occasionally goes for outreach services | *Strong collaborative environment   *Use of Protocol 1 by SOPD doctor and GOPD doctor in the community  *Use of Protocol 2 by dietitians with motivational interview technique | *Doctors are not able to adequately counsel large patient population so doctors cannot spend too much with awareness at the health centre  *Hip-waist ratio not usually done because of concern for patients' privacy | Dietician utilizes PEN protocol 2 | *Limited pace is a big issue for staff to perform their role | *Protocol 1 was used by the doctor and nurse  *Protocol 2 was used by the dietitian and physio  *Multidisciplinary approach in PEN model delivery   *Through PEN guideline, patients' management has been standardized | *Service delivery hindered by non-functional equipment  *Challenges with transportation   *Lack of counsellor limits effective health advice to patients | * Staff travelled to the community to deliver some SOPD services in order to overcome challenges of transportation in accessing health care  *Tracing of clinic defaulters conducted within the communities    *PEN has helped facility on how to appropriately scheduled follow-up for patients based on CVD risk classification | *Interruption of PEN model due to staff transfer and bringing in untrained staff.  *Lack of adequate workspace limited effective counselling, health education an food demonstration sessions  *Outreach service is limited by challenges with transportation, insufficient workers and |
|  |  |  |  |  |  |  |  |  |  |  |  |  |  |  |
| **Governance** | *PEN Audits are performed by WHO  *1 audit done | *No obvious leadership, coordination and accountability structure for PEN at the HC level  *The focus of PEN audit was on the use of the evidence-based risk tools rather than functionality of the facility | *1 PEN audit done | Not many people were able to attend PEN training at the same time due to insufficient staff  *No clear structure on how PEN equipment need to be serviced or replaced  *No designated focal persons to provide oversight for smooth day-to-day running of PEN within the facilities   *No obvious leadership, coordination and accountability structure for PEN at the HC level | *2 external audit done | *No obvious leadership, coordination and accountability structure for PEN at the HC level  *No designated focal persons to provide oversight for smooth day-to-day running of PEN within the facilities | *2 external audit done | *No obvious leadership, coordination and accountability structure for PEN at the HC level  *No designated focal persons to provide oversight for smooth day-to-day running of PEN within the facilities | *1 audit done | *No obvious leadership, coordination and accountability structure for PEN at the HC level  *No designated focal persons to provide oversight for smooth day-to-day running of PEN within the facilities | *Audit performed on medication availability at the health centre  *Audit performed and discussed with nurse on issues with use of PEN protocol  *Audit performed 6 monthly | *No obvious leadership, coordination and accountability structure for PEN at the HC level.  *No designated focal persons to provide oversight for smooth day-to-day running of PEN within the facilities | *Audit was conducted by team from Ministry according to Staff | *No obvious leadership, coordination and accountability structure for PEN at the HC level.  *No designated focal persons to provide oversight for smooth day-to-day running of PEN within the facilities |
|  |  |  |  |  |  |  |  |  |  |  |  |  |  |  |
| **Financing** | PEN activities funded by the government through the various supply chains for medication and logistics |  | PEN activities funded by the government through the various supply chains for medication and logistics |  | PEN activities funded by the government through the various supply chains for medication and logistics |  | PEN activities funded by the government through the various supply chains for medication and logistics |  | PEN activities funded by the government through the various supply chains for medication and logistics |  | PEN activities funded by the government through the various supply chains for medication and logistics |  | PEN activities funded by the government through the various supply chains for medication and logistics | *No funding allocated for the various disciplines e.g. for Dietician to make food demonstration |
|  |  |  |  |  |  |  |  |  |  |  |  |  |  |  |
| **Information** | Monthly report submitted to the headquarters through the zone nurses | No systematic way of collecting and managing PEN data |  | No systematic way of collecting and managing PEN data | Nurses, monthly report shows number of long visits they did, how many shift clinics they did, how many screen, how many newly diagnosed | *Data collection is via paper-based method  *No systematic way of collecting and managing PEN data | *Facility collect data on number of NCD cases and list of defaulters - this is sent to division and national levels | No systematic way of collecting and managing PEN data  *While colour coding is being done to risk-stratify patient, there is no data on the proportion of patient in each CVD risk class | Patients' registration and referral needs to be strengthened so they can be adequately followed up | No systematic way of collecting and managing PEN data |  | No systematic way of collecting and managing PEN data |  | *No systematic way of collecting and managing PEN data  *No data to justify if pen has really worked |
